# Drawing a line in the sand: affect and testimony in autism assessment teams in the UK

**DOI:** 10.1111/1467-9566.13063

**Published:** 2020-02-21

**Authors:** Jennie Hayes, Rose McCabe, Tamsin Ford, Ginny Russell

**Affiliations:** ^1^ College of Medicine and Health University of Exeter Exeter UK; ^2^ School of Health Sciences, City University of London London UK; ^3^ Department of Psychiatry University of Cambridge Cambridge UK; ^4^ Colleges of Medicine and Health/Social Sciences and International Studies University of Exeter Exeter UK

**Keywords:** diagnosis, autism, sociology of diagnosis, discourse, thematic analysis, UK

## Abstract

Diagnosis of autism in the UK is generally made within a multidisciplinary team setting and is primarily based on observation and clinical interview. We examined how clinicians diagnose autism in practice by observing post‐assessment meetings in specialist autism teams. Eighteen meetings across four teams based in the south of England and covering 88 cases were audio‐recorded, transcribed and analysed using thematic analysis. We drew out two themes, related to the way in which clinicians expressed their specialist disciplinary knowledge to come to diagnostic consensus: Feeling Autism in the Encounter; and Evaluating Testimonies of Non‐present Actors. We show how clinicians produce objective accounts through their situated practices and perform diagnosis as an act of interpretation, affect and evaluation to meet the institutional demands of the diagnostic setting. Our study contributes to our understanding of how diagnosis is accomplished in practice.

## Introduction

If we think about it diagnostically, somewhere there is a line drawn in the sand … and where that line is changes really, historically. (Consultant Clinical Psychiatrist)

Autism spectrum disorder (ASD) is diagnosed when there are persistent patterns of difficulty in social communication and social interaction, combined with restricted, repetitive patterns of behaviour, interests or activities (APA 2013). In a context of increasing prevalence rates, neurodiversity activism, parent advocacy, and debates around aetiology of autism, sociologists have considered how the category of autism has shifted and changed. This has included examining the role of parent activists, genetic and genomic developments, and the broader issues of psychological and child development that shape the category (Evans 2017, Eyal *et al*. 2010, Fitzgerald 2017, Hollin 2017, Hollin and Pilnick 2015, Nadesan 2005, Silverman 2013, Singh 2016).

The condition of autism is particularly interesting for a study of diagnosis because it has uncertain aetiology, symptoms can be inherently ambiguous, and rather than being a single condition, autism is considered to comprise a group of heterogeneous disorders which vary widely. Furthermore, approximately 70 per cent of people who are diagnosed are considered to meet criteria for at least one other psychiatric condition (NICE 2011). Core symptoms are considered to be present in early childhood but many people are now diagnosed in adulthood. Diagnostic rates have increased greatly, now estimated at about 1.1–1.2 per cent of the population (Brugha *et al*. 2012, Sadler *et al*. 2017), leading to debates around the broadening of diagnostic criteria (Russell *et al*. 2015, Rutter 2005); the decreasing age of diagnosis (Leonard *et al*. 2010); the conceptualisation of autism as an ‘epidemic’ (Ebben 2018) and ultimately the medicalisation of behaviour through domain expansion (Conrad 2007).

Whilst there are many screening and assessment tools for autism, the most commonly used tool in both adults and children is the Autism Diagnostic Observation Schedule (ADOS) (Lord *et al*. 2000), alongside a clinical interview such as the Developmental, Dimensional and Diagnostic Interview (3di) (Skuse *et al*. 2004) or Autism Diagnostic Interview‐Revised (ADI‐R) (Le Couteur *et al*. 2003). The ADOS is an activity‐based standardised observation tool and the clinical interview is a set of standardised interview questions for caregivers designed to elicit developmental and behavioural information to assess the presence of autistic symptoms (Skuse *et al*. 2004). Diagnosis, therefore, is determined primarily through observation of behaviours and the accounts of informants: patients,^1^ family members, friends or colleagues. The ‘gold standard’ of diagnostic decision‐making is considered to be consensus agreement within a multi‐agency team utilising appropriate diagnostic tools and other related assessments (Falkmer *et al*. 2013, Woolfenden *et al*. 2011). As a case study, therefore, there is an opportunity to consider the interaction of different agents within the diagnostic process: clinicians,^2^ patient and family testimonies, and diagnostic tools, in the institutional context of the assessment meeting.

There has been little direct observational work examining how clinicians accomplish autism diagnosis through talking about their patients together; and no studies to our knowledge that do so in adult assessment. This article explores the question of how clinicians utilise and interpret evidence together, to create an accountable diagnostic narrative.

## Constructing autism

Our study is sited within a ‘sociology of diagnosis’ framework (Brown 1995, Jutel 2009) which considers the place of diagnosis in the institution of medicine, the social framing of disease definitions and how diagnosis confers authority to medicine (Jutel 2009). Considering diagnosis as a social process rather than a ‘moment of clinical purity’ (Latimer 2013) allows investigation into the social forces that may shape diagnosis, the cultural discourses drawn upon, the ways in which human problems and experiences become viewed and treated as medical – a process known as medicalisation (Conrad 1992) – as well as the practices clinicians use together to resolve the challenge set by wider society. In this context, the category of autism can be viewed as a conceptual framework that proscribes varying contemporary versions of acceptable societal behaviour, understandings of psychiatry, as well as technological and genetic developments.

Conrad and Schneider's (1980) approach to medicalisation embraces interaction, shifting the site of medicalisation from being the responsibility of the medical practitioner alone, to a broad and inter‐related set of practices and values. At micro‐level, this can include non‐clinical actors such as teachers and employers (Halfmann 2012). The intersection of different actors (including commercial interests, advocacy groups, genetic technologies and institutions) can shift a collection of behaviours into a ‘thing’: a concrete entity which becomes a disease category. For autism, this process of reification transforms what is sometimes an inconsistent or intangible set of social behaviours into a concrete condition, perceived as an inherent attribute of an individual. Examining clinician interaction can help us to understand how social behaviours become medicalised through the social process of diagnosis.

Social and institutional practices, therefore, enable the framing of autism as a condition and as a social construct for defining the normal and the pathological. Scholars have illustrated how social practices have reconceptualised autism. For example, increased forms of social surveillance of children and the emergence of ‘childhood’ as a research focus enabled the reframing of autism as a condition in the 1940s (Nadesan 2005). Others argue that deinstitutionalisation from the 1960s led to a redistribution of expertise, a rise in parental activism, and a change in how we understand ‘mental retardation’, contributing to extending autism into a broader spectrum (Eyal *et al*. 2010). Evans argues that the development of autism in the UK must be seen in the light of major institutional transformations from the 1960s including the growth of new statistical and epidemiological methods for measuring childhood behaviour and prevalence (Evans 2013, 2017). These methods served to reconceptualise psychological development: ‘autism’ shifted from a category related to the inaccessible inner life of a child, to a problem of social impairment (Evans 2013).

The reclassification of autism as a ‘spectrum’, Autistic Spectrum Disorder, in DSM‐5 (APA 2013), simultaneously, and controversially, removed Asperger's Syndrome as a discrete and separate sub‐group. This conceptual change to a heterogeneous ‘continuity of severity’ rather than ‘discrete entities’ (Wing and Gould 1979: 26) determined that autism became a single condition with different levels of severity rather than four separate types of pervasive developmental disorders as outlined in DSM‐IV (APA 1994).

Some scholars argue that the development of the category of autism as broadly heterogeneous can be conceived as an ‘agential cut’ – a point at which autism became one thing to the exclusion of others (Hollin 2017). Hollin argues that the work of key cognitive researchers such as Frith, Happe and Baron‐Cohen in the 1980s and 1990s shifted autism into the cognitive domain as an explanatory framework. Moreover it led to a stable definition of autism ‘determined by its indeterminacy’ (Hollin 2017: 617); as a heterogeneous condition with no two people being the same, and with no single cause. Whilst there has been extensive work undertaken on medical uncertainty (see Atkinson 1984, Bursztajn *et al*. 1986, Campbell 1985, Fox 1957, Greenhalgh 2013, Hedgecoe 2003, McGoey 2009, Pickersgill 2011, Pinch 2012, Timmermans *et al*. 2018), which we discuss elsewhere (see Hayes *et al*. 2019, under review), here we consider Hollin's concept of ontological indeterminacy to be particularly relevant for this study. Hollin argues that autism's inherent heterogeneity lends it an ontological indeterminacy, meaning that exactly what autism is can never be known (Hollin 2017). Nevertheless, it still defends the status of object as a reified category.

Finally, critical diagnostic work has explored how parent advocates and adult self‐advocates have helped to shift public awareness of what autism is and how it is represented (Eyal *et al*. 2010, Silverman 2013, Singh 2016). Eyal *et al*. (2010) argue that a redistribution of expertise provided a new network whereby parents moved from being the ‘least credible’ of witnesses, for example being blamed as ‘refrigerator mothers’ (Bettleheim 1967), to experts on their children. Parents became core to a network of expertise whereby there was a ‘hybridisation of identities’ between medical experts and lay people (Eyal 2010: 8). This, they argue, means that the production of autism takes place relationally and outside the traditional medical field of psychiatry, instead in a ‘space between fields’ where boundaries between lay and medical expertise are blurred (Eyal 2010, Eyal *et al*. 2010). Combining this concept with an interactional approach to medicalisation, raises the question of how these networks of expertise work in practice, and how this might contribute towards defining the condition.

## Talk between healthcare practitioners about diagnosis

There has been some observational research examining autism assessment, for example, observation of initial assessment meetings (O'Reilly *et al*. 2017) and of multidisciplinary team meetings (Parish 2019). The empirical body of work most relevant to our study is that undertaken in the US by Turowetz and Maynard (Maynard and Turowetz 2017, 2019, Turowetz 2015a, 2015b, Turowetz and Maynard 2016, 2017, 2019). In their analysis of talk‐in‐interaction in autism assessments, case conferences and diagnostic feedback meetings, Maynard and Turowetz demonstrate how diagnostic stories are methodically produced through interaction between clinicians themselves and between the child and clinicians. This article builds on two inter‐related findings from their work related to how clinicians ‘attend to’ different factors in the diagnostic process: foregrounding diagnostically salient behaviours; and disattending to interactional agents within the assessment.

On the first point, Turowetz claim that clinicians identify and select diagnostically salient (story‐worthy) behaviours to recount to colleagues who will, together, build consensus around their importance to a potential diagnosis (Turowetz 2015b). Turowetz examined how interpretations of a child's ambiguous actions were revised and developed, and served to emphasise certain interpretive frames over others. Clinicians orient to story‐worthy events, therefore, to create diagnostic consensus (Turowetz 2015b).

Second, and with reference to Actor Network Theory (see Latour 2005), Turowetz argues that whilst behaviour in assessments is ‘interactionally‐occasioned’, clinicians cite practices in such a way that presents the assessing clinician as a neutral facilitator and diagnostic tools as largely passive recording measures (Turowetz 2015a). This finding is extended in later work where Turowetz and Maynard argue that although diagnosis is an embodied, interactional process, clinicians ‘disattend’ to interactional agents – the clinician and the diagnostic tool – within assessment (Turowetz and Maynard 2019). This is a necessity born of the institutional pressure for standardisation, and results in the behaviours being reported as an inherent feature of the child, rather than as an interaction between clinician, tool and child.

However, a UK study by Hollin and Pilnick (2018) found that the diagnostic decision can shift depending on how similar types of behaviour are interpreted. In ADOS assessment sessions, facilitated by ADOS‐trained researchers, they demonstrate how judgments are made about which kinds of behaviour are consequential for diagnosis. The authors discuss the significant level of interpretation required to identify and separate these kinds of behaviours with consistency (Hollin and Pilnick 2018). This suggests that despite attempts at standardisation, interaction and interpretation play a significant part in assessment.

Whilst Turowetz and Maynard argue that autism diagnosticians routinely ‘gloss over’ embodied interaction in assessment (Turowetz and Maynard 2019: 1023), other researchers have shown how affect, emotion, and the expert ‘gaze’ are woven into the social actions of different stakeholders in the world of autism (e.g. Fitzgerald 2013, 2017, Hollin and Giraud 2017).

Silverman (2013) argues that autism as a condition is characterised by expert knowledge, through standardised systems of measurement and description with their associated screening and diagnostic tools. And yet, for parents and families these standardisations fail to represent the emotional consequences of these diagnostic practices, or the messiness of real life experience (Silverman 2013). Silverman argues that, rather than being a liability, emotion can be a source of committed and reliable knowledge. Fitzgerald extends this argument to suggest that autism neuroscientists engage in their work not simply as an intellectual or technical task but as an act of affective and emotional labour (Fitzgerald 2013, 2017). In his interviews with neuroscientists he finds that they express a ‘feel’ of autism, as something distinct and knowable, as something we ‘recognise when we see it’ (Fitzgerald 2017: 48). He argues that, where specificity in testing is less important than the level of impairment, ‘epistemological space’ is given to whether autism is ‘felt’ by the clinician during the interaction (Fitzgerald 2017: 50).

These empirical studies serve to illustrate how clinicians may navigate the complex process of diagnosis when heterogeneity is considered to be core to the condition. In this article, we focus on the way in which clinicians talk about diagnosis together in specialist autism assessment meetings in the UK. We were particularly interested in how diagnosis of autism is constructed as an interactive event within meetings and how this might contribute to the reification of autism as a condition.

## Methods

As part of a larger study exploring autism diagnosis, this study collected naturally occurring data by observing how clinicians talk together in specialist autism assessment teams. Using naturally occurring data in healthcare settings enables exploration of real‐world practices underpinning health care (Kiyimba *et al*. 2019). It provides data which is local and contextually focussed and therefore ‘context‐rich’; and minimises the active role of the researcher in shaping the data (Kiyimba *et al*. 2019, Potter 2002).

We purposively sampled teams that specialised in autism assessment and who held regular assessment meetings. Four teams took part in the study: two adult, and two children and young people (C&YP) assessment teams. Patients and families were not present at any meetings.

Sites were recruited from an open call to a list of clinical contacts drawn from the internet and via the Institute for Health Research (NIHR) Clinical Research Network. All sites were National Health Service providers. Seven teams were approached: two were excluded as they did not hold formal meetings, and one withdrew. Table 1 gives characteristics and description of each team by role and setting information.

**Table 1:** Team characteristics and diagnostic outcome

Team details^a^	Meeting number	Attendees per meeting	Total case discussions	Diagnosed with autism	Diagnosed not autism	Deferred^b^	Referrals & admin discussions^c^
Adult Team 1^d^	1	6	3	1	0	2	0
Clinical Team Manager; Clinical Psychologists (×4), Assistant Clinical Psychologists (×3); *Peer Support Worker*. Meets weekly (case review meetings) predominantly rural area	2	6	2	0	0	2	0
	3	5	1	0	0	1	0
	4	6	2	0	0	2	0
	5	5	3	0	0	3	0
			**11**	**1**	**0**	**10**	**0**
Adult Team 2	1	5	3	2	1	0	8
Team Manager; Consultant Psychiatrists (×2); Occupational Therapist; Medical Secretary; *Social Worker*;* Speciality Doctor*;* STS Psychiatrist*. Meets weekly; Predominantly urban area	2	4	6	2	1	3	6
	3	5	5	1	2	2	5
	4	4	2	1	0	1	5
			**16**	**6**	**4**	**6**	**24**
CYP Team 1^d^	1	6	9	3	3	3	4
Clinical Team Manager; Consultant Psychiatrists (×2); Clinical Psychologist; Senior Manager; Speech & Language Therapist; Educational Psychologist; Autism Practitioner (×2); Senior Administrator. Meets monthly with ‘mini’ meetings as required; Predominantly rural area	2	5	2	2	0	0	4
	3	4	1	0	0	1	2
	4	4	6	4	0	2	1
	5	3	2	1	0	1	2
			**20**	**10**	**3**	**7**	**13**
CYP Team 2 (14 + )	1	2	1	1	0	0	0
Consultant Psychiatrist; Clinical Psychologist (×2); *Clinical Psychologist*;* Senior Nurse*;* Foundation Doctor*. Meets weekly; Predominantly urban area	2	3	1	1	0	0	0
	3	4	1	1	0	0	0
	4	4	1	1	0	0	0
			**4**	**4**	**0**	**0**	**0**
Totals			**51**	**21**	**7**	**23**	**37**

a Not all team members were present in every meeting; *visitors in italics*.

b Cases were deferred due to team requiring further tests or information.

c Cases which came in as referrals and were allocated for assessment: or were subject to admin discussions.

d In these teams only selected cases were brought for presentation.

A process of ethical approval and research governance was undertaken in line with NIHR Good Clinical Practice guidelines. This included full informed consent from participants, rights to withdraw from the study, secure storage and data management. Names and identifying details were changed throughout to protect the identity of participants and patients. Ethical approval was granted by University of Exeter Medical School ethics committee (Ref: Mar17/B/114) and the Health Research Authority (Ref: 220180).

### Data collection

Observations of meetings were carried out by the first author who audio‐recorded 18 autism specialist team meetings in four different sites (nine meetings in two adult and nine in two C&YP settings). We used Malterud *et al*.'s (2016) concept of ‘information power’ to assess when we had adequate data to meet the aims of the study. This involved consideration of quality of dialogue, analysis strategy, use of established theory and sample specificity.

The number of cases discussed at each meeting ranged from 1 to 9, and in total the observations provided data related to 88 cases and documented over 19 hours of meeting time. Thirteen cases discussed were at referral stage and 24 were classified as administrative discussions, primarily related to booking dates for assessments or related actions. These two groups were excluded from analysis as they did not involve decision‐making about diagnosis. The final analysis included a total of 51 cases – 24 children and young people and 27 adults. The first author's field notes, short case summaries written after fieldwork and the transcribed, anonymised audio data comprised the dataset.

### Method of analysis

We undertook a thematic analysis which enabled identification of patterns across the dataset (Braun and Clarke 2006, 2013). Thematic analysis is theoretically flexible (Braun and Clarke 2013) and we took a social constructionist approach which acknowledges that meaning is socially produced and reproduced rather than being located within the individual (Burr 1995). This social constructionist approach makes the epistemological assumption that we create meaning collectively through our interactions and experiences of the world, and that our constructions of reality depend on specific cultural and historic factors (Burr 1995). Knowledge is produced, therefore, by and through culture, language and discourse. Discourse is considered to be a social practice with consequences (Edwards and Potter 1992) and by examining clinicians’ talk, we can examine how ‘facts’ and meaning are socially produced.

Our analysis was based on a data‐driven, inductive, organic approach (Clarke and Braun 2018) prioritising depth of engagement. We followed Braun and Clarke's six‐stage process: familiarisation, coding, generating, reviewing and defining/naming themes, and writing up (Braun and Clarke 2013). Throughout, we focussed on discursive issues as outlined by Wiggins (2017), which allowed the authors to consider how, through interaction, the social action of diagnosis is accomplished.

Data were transcribed orthographically, in line with thematic analysis, and initial coding was undertaken by the first author across the complete dataset using Nvivo software (QSR International, Melbourne, Australia). The developing analysis was conducted by the first author, presented at qualitative data sessions and discussed with co‐authors throughout to develop and challenge emerging ideas and to develop consensus.

## Analysis

Clinicians considered a range of assessment material in their meetings that included the results of diagnostic tests, primarily the ADOS and a clinical or developmental interview such as the 3di. The team discussion led either to a diagnostic decision – autism or not; or a deferred decision dependent on further testing or information (Table 1). In the process of analysis, two inter‐related themes were identified in relation to the way in which clinicians expressed their specialist disciplinary knowledge in order to come to diagnostic consensus: Feeling Autism in the Encounter; and Evaluating Testimonies of Non‐Present Actors.

### ‘Feeling’ autism in the encounter

We found that clinicians routinely expressed an idea that they could feel or sense when a person they were assessing was autistic.

Cameron is a 9‐year old boy. The Speech and Language Therapist (SLT) has undertaken the ADOS assessment and has ‘*scored*’ Cameron as within the clinical range suggesting autism. However, there is concern that there may be other factors that might explain his score, due to a complex family history. There is an impasse in the discussion and the Clinical Team Manager (CTM) prompts a shift in direction.

**CYP Team 1: Cameron, age 9**

CTM You've got a feeling that it felt ASD?

SLT Oh yeah.

CTM Yes, which you can tell by the way you're reporting it

The question, framed as a statement, offers SLT an opportunity to underpin the ADOS report with a subjective impression. There is a shared understanding that this subjective account has weight in this setting; it makes the clinician doubly accountable for assessment, both ‘objectively’ through the diagnostic tool, and ‘subjectively’. Our interest is not whether or how SLT ‘feels ASD’: rather what the expression of ‘feel’ might be doing discursively. There is invocation of two types of feeling here. The clinician has a feeling; and in addition ‘it’ felt ASD. The use of ‘it’ rather than ‘he’ is ambiguous in relation to the location of this aspect of ‘feeling’; the way in which this is framed sites the feeling not in the child, but somewhere else, in the space between.

This can be seen further in discussion about Gail, where the location of the feeling is ‘*in the room*’. Gail ‘*didn't quite reach threshold*’ on the ADOS, suggesting not autism, but the Clinical Psychologist (CP) and an Assistant Psychologist (AP) are pursuing further tests.

**Adult Team 1: Gail, age 44**

AP I think though we're just going to do some more with her aren't we? I don't feel like there's nothing in it.

CP Absolutely. I think there is something in there and I think it's that having to remember what it was like being in the room with her because … and I think particularly I'm finding it quite helpful to talk about her and actually, because I'm quite cynical about certain aspects

Here clinicians recall the experience of being ‘*in the room*’ with Gail and this appears to be a motivator for pursuing ‘*some more*’ (tests). The participants agree that there is ‘*something*’ and this is driven by feeling and ‘*what it was like*’. Here we do not assume a cognitivist position which asserts that either AP or CP are *experiencing* emotion during this process of assessment. Neither do we claim that these things are *not* being experienced. Rather, we consider this expression of affect as a social action rather than a reflection of inner states. Here clinicians are accounting for their continued pursuit of diagnosis, despite a score on the ADOS that does not indicate autism, and some cynicism about the narrative being told by Gail and her informant. Like the ‘affective space’ (Fitzgerald 2014: 245) within which autism scientists work, here ‘feeling’ circulates dynamically and expansively between actors, its transitioning presence enabling discursive flexibility and warranting future action.

‘Feeling’ can be seen to drive forward assessment. Brian is a 58‐year‐old man who was assessed by a CP and was scored ‘*under‐threshold*’ on the ADOS, outside the clinical range for autism. However, the clinicians in assessment considered the case further.

**Adult Team 1: Brian, age 58**

CP It's just reminding me a little bit of who we saw today in that we both feel there's something in it but we don't feel we've quite got to it yet and it's something around sometimes how you get there and how you ask these questions. It's uncovering it, isn't it? It sometimes takes a little while to get there as well

CP suggests that there is ‘*something*’ inherent to Brian that requires a particular kind of approach to ‘uncover’. This locates the source of the condition, autism, as embedded within Brian and asserts the special knowledge of the clinician to discover it. Clinicians’ stated ability to ‘know’ there is something there, a clinical intuition, is a kind of ‘disciplinary objectivity’ (Timmermans and Buchbinder 2012) – a specialised evaluative knowledge or trained judgement (Daston and Galison 2007) – which can drive clinicians to seek further evidence towards a diagnosis or support a particular diagnostic trajectory. However, as with Cameron and Gail, the location ‘it’ assumes there is a ‘thing’ that has to be found. The CP's analysis suggests corroboration with the second assessor with the use of ‘we’, thereby strengthening the account and their accountability. Here we can see what Fitzgerald (2014: 235) calls a ‘particular dynamic of ambiguity and presence’ in that there is a certainty and commitment embedded in the expression of the feel of autism, and yet the expression of it emerges from ambiguity in assessment. Finally:

CP And essentially that was kind of driven by the fact that ultimately we kind of feel that there's something there actually but he's not necessarily playing ball and reporting it or its not necessarily coming up in the way we'd expect

Brian's lack of co‐operation or insight (‘*not necessarily playing ball*’) is invoked as a reason why autism may not be apparent when the clinicians believe there is ‘something there’. The process leads to a ‘firm conviction’ (Messer *et al*. 2018: 268) as the final guide to diagnosis which allays any uncertainty previously experienced when Brian demonstrates insight into his own difficulties in a feedback meeting. Brian was diagnosed with autism.

CP I felt that he met criteria and, what's more, I felt for the first time I felt actually that there was sort of clinical grounds for the diagnosis

Unlike some other instances where clinicians cited various forms of evidence, here the clinician describes their own judgement or ‘feel’ for autism. In the space of the team meeting, expressions of feeling serve to drive or inhibit progress towards a diagnostic decision, makes diagnosis doubly accountable, and allows the expression of dilemmas and contradiction. It becomes a warrant for further exploration. Whilst the *feeling* resides between assessor and assessed, the *behaviour* is located in the individual being assessed. And yet, as noted by Hollin and Giraud (2017), affective responses are not entirely determined by the organism (patient) but by the ‘whole ecological setting within which that organism is immersed and perceived’ (Hollin and Giraud 2017: 2). Clinicians are *part of* the ecology of diagnosis, not separate to it. The discursive accomplishment of expressing affect is to signal a shared understanding of what autism ‘feels like’ to clinicians who are also part of that ecology and who, in accepting this affective assessment, jointly make ‘feeling’ relevant to the task in hand. The expression of affect, therefore, enables collective ‘interactional progressivity’ (Maynard and Turowetz 2017: 265) towards a decision.

### Evaluating testimonies of non‐present actors

Throughout the meetings clinicians cited instances of behaviour reported by non‐present patients, their parents, partners or other informant, frequently drawn from a clinical interview such as ADI‐R, which tacitly or explicitly linked to diagnostic criteria for autism.

Elisha is a 15‐year‐old girl who has been assessed by clinical interview (3di) and ADOS. Elisha has ‘*scored up across all domains*’ on the 3di, suggesting that she is within the range for an autism diagnosis. The CP suggests that she is also likely to ‘*score up*’ on the ADOS, which has not yet been scored. Here Consultant Psychiatrist (CPi) recounts a story told by Elisha's parents.

**CYP Team 2: Elisha, age 15**

CPi The other unusual thing she started doing at that time was when she left her home to visit her friends, she would insist on bringing all of her clothes with her on the bus, which I think the parents went along with, although it was such hard work bringing these clothes back and forth

Firstly, CPi primes listeners to hear the upcoming talk in the context of abnormality by prefacing the story with ‘*the other unusual thing*’. ‘*She would insist*’ suggests that Elisha's actions were undertaken in the face of opposition, presenting her as unreasonable. The parents then have to engage in ‘*hard work*’ which indicates significant effort required to meet Elisha's needs. Together, the elements of this narrative suggest a young person whose behaviour is troublesome, with the speaker inviting listeners to locate symptoms of autism – restrictive, repetitive behaviours, interests or activities – in Elisha's behaviour. Here CPi is actively constituting parental reports about Elisha's behaviour as evidence of autism and drawing on ‘the importance of parents’ caring as a source of insights’ into their own children (Silverman 2013: 229). Parent testimonies are valued and reinforced by clinicians in their retelling, with the parents’ commitment to their child (‘*went along with*’, ‘*hard work*’) and parental understanding of the difficulties of Elisha's behaviour invoked. Alongside the reporting of many such instances, observations made during the ADOS, test scores and discussion of potential complicating co‐conditions, the clinicians agree a diagnosis of autism for Elisha, on condition that the ADOS scores as expected.

With Hayley, who is 16, the ADOS assessor, Senior Manager (SM), does not undertake a full scored ADOS stating, ‘*I wasn't quite sure how she [Hayley] was going to react*’ and instead used the ADOS guidelines for a conversation with Hayley. SM recounts a number of descriptive incidences, both ‘tendencies’ and ‘instantiations’ of autistic behaviours, as defined by Maynard and Turowetz (2017), to support the assessment.

**CYP Team 1: Hayley, age 16**

SM Mum said that she used to twirl when she was younger … Em, always been obsessed with various things … and this is come from Hayley: Only Fools and Horses, Finding Nemo, em Monsters Inc, The Simpsons, Ghostbusters

SM presents this as mum's narrative, with the term ‘*she used to*’ suggesting a behaviour in the past (and no longer observable, although here cited as developmental information towards a diagnosis); and ‘*always been obsessed*’ as a recurring and ongoing difficulty. Presenting interests as an obsession constitutes Hayley's behaviour as problematic and unusual rather than as constituting normal childhood interests. Listing a large number of interests reinforces, through illustrative detail, the repetitive behaviour and adds rhetorical strength to the argument (Wiggins 2017). Here SM has recounted stories told by both Hayley and her mum. Although the two stories seem apparently disconnected, they combine to enable SM to present Hayley's behaviour as aligned with autism symptoms – restricted and repetitive behaviours – and actively constitute her behaviours, both past and present, from two different sources, as evidence of autism. Together, the elements of this narrative suggest a young person whose behaviour is unusual, with the speaker inviting listeners to locate symptoms of autism in Hayley's behaviour. Hayley is diagnosed with autism.

In both Elisha and Hayley's cases, clinicians construct the accounts of patients and families as warranted and plausible evidence of autism. Clinicians can be seen to ‘narrate objectivity’, that is, to construct the behavioural accounts of patients and families as objective evidence (Timmermans and Buchbinder 2012) and use their disciplinary expertise to build those accounts as evidence of autism. Therefore, behaviours which might be unusual are embraced with a category of autism, and reproduced through talk as what autistic behaviour looks like.

Here lay and professional expertise interact: from a lay perspective, patients and families understand what constitutes autistic behaviour and utilise it within the assessment process, whilst clinicians use their specialist disciplinary perspective to weight the value of this parent/patient testimony. However, in the process of corralling lay testimonies in the team meeting, there is a blurring of boundaries (Eyal 2010) between the source and nature of that expertise. Whilst, as Eyal argues, the expertise of parents was once interpreted as ‘parental coldness’, or evidence of the cause of the condition, the ‘credit‐worthiness’ (Eyal 2010: 4) of that expertise now has been upgraded to be almost indiscernible from clinicians’ own accounts. However, as we illustrate here, clinicians also distance themselves from patient and family accounts as evidence, when they are considered un‐creditworthy.

In some cases patients were considered by clinicians to have researched or rehearsed relevant behaviours, leading to them ‘perform’ autism. We return to Gail, who has come to assessment with a friend who has been interviewed as Gail's ‘informant’. The team is worried that Gail is too invested in receiving a diagnosis.

**Adult Team 1: Gail, age 44**

CP This sounds also like I mean we've talked about this quite a lot haven't we, but in those situations where someone comes and they are quite invested, there is a … sort of slight recoil … and that I think is what I'm struggling with. It gives you a sort of even more sceptical edge I think sometimes doesn't it?

CP acknowledges that a perception that a patient is ‘invested’ in diagnosis can impact on how they, as clinicians, ‘feel’ about the patient and their diagnosis. This is further developed when the interview leads to a conclusion that the friend is keen for Gail to be diagnosed too and ‘*sees herself as a bit more of an expert in autism*’. The team question how much Gail and her friend have planned the assessment together.

AP It's hard to not get a word in edgeways, but kind of get to the bottom of actually how rehearsed is this? How much have you spoke about this? How much have you researched this? It felt very much like none of the questions I asked were a surprise in anyway

AP expresses mistrust of the patient/informant account, casting doubt on its veracity. A consideration of motivation and ‘investment’ contributes to judgement of the credibility of the patient's position and consequently the weight clinicians give to those reported behaviours. Patient accounts that are too coherent (or too chaotic in other cases) raise suspicion. In Gail's case, the lead clinician also felt there was ‘*something*’ there and the case was deferred pending further observation and interviewing a second informant.

Finally, Nadia is a 29‐year‐old woman who has been assessed by the Team Manager (TM) whilst in hospital. TM is concerned that, despite scoring ‘quite high’ on the ADOS, suggesting autism, the result may be invalid as Nadia was dosed on heavy medication and this ‘*affects a lot of your interaction with her*’. TM then also expresses concern to the CPi that Nadia may be invested in diagnosis:

**Adult Team 2: Nadia, age 29**

TM And then she said at one point, she said to me, cos she wants the diagnosis, … ‘oh what would an autistic person say?’

CPi That's really interesting. What would she gain from an autism diagnosis? Like why would she want it?

TM They've told her unhelpfully … that we're going to see her and we're going to fmd her a great new house.

TM introduces this incident as an interjection in a number of problematising factors about the assessment. TM prefaces this story with ‘*cos she wants a diagnosis*’ which alerts the listener to hearing the story as someone motivated to manipulate the assessment. ‘*They've told her unhelpfully*’ suggests that TM thinks that the assessment has been compromised, because a clinician has misinformed Nadia who then has misconceptions about the potential benefits of a diagnosis, leading to her attempting, albeit unsuccessfully, to ‘perform’ as autistic in assessment.

In this case a patient was perceived to be attempting to manipulate the assessment for secondary gain thereby alerting clinicians to question the credibility of the patient. Here Nadia's account is used to construct a case against an autism diagnosis, constituting Nadia's behaviour as manipulative rather than autistic. The result for Nadia was left open as she was still to be assessed through a clinical interview with the CPi.

To summarise, in the cases of Hayley and Elisha, the ADOS is downplayed in favour of parent and patient narratives which support autistic behaviours. Parental accounts align with the anticipated outcome and are constituted as autistic behaviours both by parents and by clinicians. With Gail and Nadia, the ADOS is compromised or questioned due to the testimony or behaviour of the patient in assessment that suggests that they might be trying to subvert the assessment.

In these cases, clinicians use their disciplinary expertise to assess the veracity and value of lay expertise. Their collective knowledge determines that patients can manipulate assessment for secondary gain; that they can perform or rehearse autism if invested in diagnosis; and that lay knowledge of autism can help or hinder, depending on its credibility. Clinicians decide the value of different accounts to offer a warrant for diagnosis or not.

The discursive accomplishment of co‐opting patient and family accounts is to enhance (or diminish) the trajectory towards diagnosis. Reported speech in particular can serve to enhance the factuality of the account (Wiggins 2017) and the extent to which the patient is presented as agentic (e.g. Elisha cannot help her troublesome behaviour but Nadia is motivated to adapt her behaviour for secondary gain) works discursively to present the patient/family as credible or not.

## Discussion

Our study builds on Turowetz and Maynard's (2019) work examining autism diagnostic practices *in situ*. We have found, in line with these researchers, that clinicians foreground discussion of behaviours that are ‘story‐worthy’ and these include both their own observations as well as harnessing informant testimony that contribute to the diagnostic account. We also found, in line with Hollin and Pilnick's (2018) study, that, in the process of retelling the behavioural story, clinicians interpret that behaviour in the light of autistic behaviours.

We found that clinicians attended to the embodied impact of ‘being in the room’ with the patient. Like Fitzgerald (2017) and his neuroscientists, clinicians routinely invoked the qualitative description of ‘feeling’ an interaction as autistic. Clinicians directly referred, therefore, to their own interaction with the patient, although this was not apparently reflective of their role as an active agent impacting on the patient's behaviour, rather it served to reiterate the location of autistic behaviours within the patient. The behaviour of the patient, however, can affect the clinician's approach to standardisation, for example, when the assessor changes the assessment to allow for anticipated patient behaviour (Hayley); or when there is a recognition that other factors (e.g. medication) can impact on assessment. We have not found clinicians to be naïve to the social complexities of their job, indeed, they appear to be acutely aware of the difficulties, ambiguities and social consequences of their task.

In this context, clinicians are permitted to draw on their disciplinary expertise (unlike ‘lay experts’ whose sense of ‘feel’ can only ever be opinion; or beyond this diagnostic space, where clinicians, socially, would not be warranted to ‘diagnose’). It is not, therefore, that the clinician always takes for granted (and therefore renders invisible) their own role in the assessment process as argued by Turowetz and Maynard (2019). Rather, by institutional necessity, and due to indeterminacy, they stake their own expertise on what happens in the social space between patient and clinician. This serves to enable dissent from the ‘objective’ evidence of the diagnostic tool, to bring colleagues on board with this dissent, and to re‐align the evidence in this new embodied context. It presents clinicians, not as unknowing slaves to criteria, but as active agents in the diagnosis; as one human being in relation to another. In the space of indeterminacy, clinicians draw, not just on references to where symptoms can be seen in the patient, but to affect; to how the patient in this room, at this time, makes them feel.

When clinicians invoke feeling they are staking out their territory. Feeling here is not presented as either a lack of neutrality or a call to subjectivity: it is presented and received as objectively important, as a different kind of objectivity. This assertion of an autistic presence is received as concrete and objective knowledge by colleagues. The specialist disciplinary understanding, or expert gaze (Featherstone *et al*. 2005) determines that there is ‘something there’ and is therefore treated as a source of committed and reliable knowledge. It asserts credibility on the part of the clinician, as the only way they can feel autism is through having experienced it many times.

Drawing on Eyal's exploration of networks of expertise, we found that clinicians interact with different types of expertise in an applied and pragmatic way to achieve the function of the institution: diagnosis. In team meetings, clinicians draw on both their own disciplinary expertise, and that of patient and family actors, who are absent but discursively present (Clarke and Star 2008), to both understand and shape concepts about autism. Patients and families (informants) are considered by clinicians to ‘know’ autism, not just from experience, but as ‘well‐informed citizens’ (Schutz 1946), or active agents, able to understand and construct a body of ‘story‐worthy’ evidence for presentation. Informants, in their desire for diagnosis, retell their testimony knowingly. This form of lay expertise lies not in their experience of particular behaviours alone, but in the contemporary meaning society prescribes to those behaviours, and their access to diagnostically relevant information and cultural frames.

Our study shows that in the absence of the body of the patient, the clinician recalls story‐worthy events in their stead, their presence retold through descriptive instances of pertinent behaviours that can be seen as troublesome. Testimonies (motivated, mediated, partial, variously‐informed, invested, interpreted and bodily absent), and how clinicians feel about them, are core to assessment. Here diagnosis is happening relationally, with the absent voices of patients and families being interpreted by the clinician. As argued by Eyal (2010) we can see how medical and lay expertise is blurred in the discussions between experts: the knowing patient narrative is re‐constructed through the medical lens of autism. However, clinicians acknowledge together that informants have stake and interest, and the veracity and value of their testimony is judged according to the clinician's expert understanding. Although this might be indicative of Eyal's (2010) ‘space between fields’, the way in which expertise is drawn on is, inevitably, asymmetrical (Pilnick and Dingwall 2011).

The process of diagnosis is ‘institutionally determined’ (Gill and Maynard 2012) in that the rhetorical ‘allowance’ in the clinic is, by necessity, for a diagnosis of autism or not: the delivery of this binary decision is the institutional imperative and must be the key task of the team. But diagnosis is socially framed in the clinic, and is subject to practices and processes of debate and adjudication between different forms of evidence (Latimer 2013). Clinicians accomplish diagnosis by necessity, constructing objectivity from indeterminacy via discursive resources, through affective, interpretive and evaluative labour. Testimonies of patients and families are co‐opted as evidence; diagnostic tools are interpreted in the light of disciplinary objectivity (and also narrated in this way); and disciplinary objectivity in the form of expressions of affect can serve to support or disrupt the diagnostic momentum.

The institutional imperative across the institution of medicine is to diagnose. We argue that the findings here related to autism diagnosis have broader relevance to understanding diagnosis more generally. As an act of affect we have explored the way in which clinicians translate inter‐subjective feeling into a diagnostic outcome. A recent review of literature suggested that a ‘Praecox Feeling’ of ‘bizareness’ is a determinant in medical decision‐making in schizophrenia (Gozé *et al*. 2019). Despite attempts at standardisation, therefore, affect can be seen an active presence in diagnostic deliberations – a signal for reflection, further exploration or testing. As an act of interpretation and evaluation we have considered the role of patient and family testimonies in diagnostic practice. This indicates how parental and family knowledge has been formalised and incorporated into medical knowledge through the adoption of the clinical interview as a standardised test. Lay expertise and the role of the family as ‘credible witnesses’ are central in diagnostic deliberation. Acknowledging and examining inter‐subjectivity in diagnostic practice, therefore, seems extremely pertinent across conditions and medical practices, as, rather than constituting a linear clinical practice, it illuminates the process of diagnosis as relational and interactively constructed between patient, family, clinician and diagnostic measure.

### Strengths and limitations

This study is one of few to directly observe clinician interaction in diagnostic decision‐making, particularly in closed team meetings. This makes the data rich and relevant to contemporary understandings of diagnosis. Our current analysis represents a broad brush‐stroke of our data: interaction in assessment meetings is complex and the content of discussion is wide‐ranging. Here we endeavour to consider key threads through the data, each of which might be expanded on further. There is a need to examine all stages of assessment, particularly those that take place in informal interactions and in the presence of patients and families.

## Concluding comments

Our study adds to the growing literature on sociology of diagnosis by furthering our understanding of how diagnosis is accomplished in practice. We argue that autism as a condition is, in part, shaped through this clinical interaction, through the interpretation of behaviours as framed by patients and families; and through a sense of autism as a ‘thing’ that can be experienced by clinicians. Schrader (2010) argues that what we know cannot be separated from the way that we know it. Autism is an object of knowledge: it is what we know, but it is an object delineated by the process of knowing it. In clinicians’ talk, autism is rendered an object *through the process of* its identification by healthcare practitioners. Uncertainty inherent in autism's heterogeneity of presentation and aetiological variation (what Hollin (2017) refers to as autism's ‘ontological indeterminacy’) is dismissed and re‐interpreted in diagnosis in order to reify autism, as fixed, real and knowable.

Reification is the institutional requirement for these diagnostic services. Clinicians understand that autism is a pragmatic psychiatric construct, underpinned by best evidence but still somewhat indeterminate, but nevertheless they must act to reify, as their role necessitates the assignation (or not) of a diagnosis. They actively constitute informant stories as evidence of autism and constitute symptoms through and in their deliberations. The net effect is to present autism as an ontologically ‘natural kind’ (Hacking 2007, Verhoeff 2012), with autism diagnosis ‘validating a reality’ (Jutel 2009). This confirms a particular kind of person as autistic and the category becomes reified (Hacking 2000). Objectivity is produced through these situated practices.

## Data statement

Access to the data that support these findings will be granted on request to the corresponding author via UK Data Service at http://reshare.ukdataservice.ac.uk/.

## Notes

1 There is significant debate about classifying autism as a ‘disorder’, with many preferring to use the term ‘condition’ (NICE 2012). We use the term ‘autism’ to embrace the spectrum of conditions as currently defined in the Diagnostic and Statistical Manual of Mental Disorders, fifth edition (DSM‐5) and International Classification of Diseases, 11th revision (WHO 2018).

2 We use the term ‘clinician’ to encompass all healthcare participants in this study, defined as members of a registered health profession involved in direct patient care.
